# Advance of Serum Biomarkers and Combined Diagnostic Panels in Nonalcoholic Fatty Liver Disease

**DOI:** 10.1155/2022/1254014

**Published:** 2022-06-29

**Authors:** Yuping Zeng, He He, Zhenmei An

**Affiliations:** ^1^Department of Laboratory Medicine, West China Hospital, Sichuan University, Chengdu, China; ^2^Department of Endocrine and Metabolism, West China Hospital, Sichuan University, Chengdu, China

## Abstract

Nonalcoholic fatty liver disease (NAFLD) affects approximately 25-30% population worldwide, which progresses from simple steatosis to nonalcoholic steatohepatitis (NASH), fibrosis, cirrhosis, and hepatocellular carcinoma, and has complications such as cardiovascular events. Liver biopsy is still the gold standard for the diagnosis of NAFLD, with some limitations, such as invasive, sampling deviation, and empirical judgment. Therefore, it is urgent to develop noninvasive diagnostic biomarkers. Currently, a large number of NAFLD-related serum biomarkers have been identified, including apoptosis, inflammation, fibrosis, adipokines, hepatokines, and omics biomarkers, which could effectively diagnose NASH and exclude patients with progressive fibrosis. We summarized serum biomarkers and combined diagnostic panels of NAFLD, to provide some guidance for the noninvasive diagnosis and further clinical studies.

## 1. Introduction

Nonalcoholic fatty liver disease (NAFLD) has recently been proposed to rename metabolic-associated fatty liver disease (MAFLD) [1, 2]. The diagnosis criteria have changed from “exclusive,” that is, excluding liver steatosis caused by excessive alcohol intake, virus, drugs, etc., to “definitive,” that is, the existence of evidence of liver steatosis (imaging, serum biomarkers, or histopathology) and the combination of overweight/obesity, type 2 diabetes (T2DM), or metabolic dysfunction [1, 2]. This renaming emphasizes the diagnostic value of metabolic disorders, such as obesity and diabetes, in NAFLD. However, related renaming is controversial, so this paper continued the traditional nomenclature to describe the disease.

With the improvement of lifestyle, the prevalence of obesity increased yearly [3], including related comorbidities, such as T2DM, hyperlipidemia, NAFLD, and metabolic syndrome (MS). Overweight patients account for about 1.5 million worldwide, and up to 90% of obese patients have a combination of NAFLD [4]. Research has reported that T2DM is one of the strongest risk factors for NAFLD [5]. NAFLD could progress from simple steatosis (SS) to nonalcoholic steatohepatitis (NASH) and even to fibrosis, cirrhosis, and end-stage hepatocellular carcinoma (HCC) [6, 7]. NAFLD increases twofold higher risks of metabolic syndrome/diabetes and is independently associated with cardiovascular events and extrahepatic complications [5, 8]. With the pandemic of obesity, the number of NASH-related death will increase by 178% by 2030 [9], becoming the most common cause of liver-related death in the future. Therefore, there is an imperative need for early screening, early diagnosis, and early treatment of NAFLD, to reduce the disease burden and socioeconomic pressure.

At present, the gold standard for the diagnosis of NAFLD is liver biopsy, which is an invasive operation that has the risks of bleeding, pain, and death, sampling deviation, poor representation, pathologist's empirical judgment, and poor patient compliance [10]. Clinically, the diagnosis of NAFLD mainly depends on ultrasound. However, ultrasound has a low sensitivity in obese patients and is not suitable for large-scale population screening [10]. Additionally, NAFLD could be reversed early on with lifestyle intervention or physical activity. Nevertheless, the current difficulties in the early diagnosis of NAFLD limit the implementation of treatment plans and miss the best time for treatment. Therefore, it is crucial to investigate early noninvasive diagnostic biomarkers and monitor the disease progression of NAFLD. This review summarized the advance of serum biomarkers and combined diagnostic panels in the diagnosis and staging of NAFLD, to provide some guidance for the noninvasive diagnosis and further clinical studies.

## 2. Apoptosis Biomarkers

The most studied NAFLD-related serum biomarker is the hepatocyte apoptosis product, cytokeratin-18 (CK-18) [11, 12], accounting for about 5% of liver proteins [13]. The sensitivity (SEN) of CK-18 M30 and CK-18 M65 for the diagnosis of NASH was 70% and 63.6%, the specificity (SPE) was 83.7% and 89.4%, and the area under the curve (AUC) was 0.71 and 0.81, respectively, indicating an excellent diagnostic value of CK-18 in NAFLD [14]. However, for patients with progressive fibrosis, the SEN of CK-18 was only 54%, with an SPE of 85% and an AUC of 0.68 [15], suggesting insufficient SEN of CK-18 for monitoring the fibrosis progression; therefore, CK-18 should be utilized in combination with other biomarkers (Table 1).

Chuah et al. [16] found that the combination of CK-18, aspartate aminotransferase (AST), and homeostasis model assessment (HOMA) (MACK-3) significantly improved the accuracy of the diagnosis of NASH. The SEN was 84.2%, the SPE was 81.4%, and the AUC was 0.81. Gao et al. [17] combined MACK-3, MS, and platelets (PLT) to establish a nomogram, which improved the diagnostic efficacy in patients with progressive fibrosis, with an NPV of 94.7%. The combination of CK-18, ALT, and MS had an AUC of 0.88 for the diagnosis of NASH in obese subjects [18]. Thus, these studies suggested that CK-18 could be an effective diagnostic biomarker for NASH, especially in combination with other biomarkers. These combined diagnostic panels could monitor the disease progression of NAFLD.

## 3. Inflammatory Biomarkers

Excessive accumulation of triglycerides (TG) in hepatocytes could further develop into ballooning, inflammation, and fibrosis. TG content [29] did not correlate with the severity of NAFLD, while the precursors or intermediates such as palmitate, diacylglycerol, and ceramide could cause mitochondrial dysfunction as well endoplasmic reticulum stress, resulting in hepatocyte damage and release of proinflammatory cytokines. Therefore, inflammatory factors are possible diagnostic biomarkers for patients with NASH. Currently, alanine aminotransferase (ALT) and AST are used clinically as biomarkers of inflammatory damage in hepatocytes. For NAFLD patients undergoing bariatric surgery, ALT and AST could be used to monitor disease progression and assess the clinical benefits of treatment strategies [30]. However, NAFLD patients could also exhibit normal ALT levels and even decrease in patients with progressive fibrosis [31], suggesting that ALT alone cannot be relied upon to determine the severity of NAFLD. Several studies have proposed that combined diagnostic panels using routine indicators could effectively diagnose NAFLD [32, 33] (Table 2). All of these models have high diagnostic accuracy and are routinely available clinically, but the specific cut-offs need to be further optimized.

Golgi protein 73 (GP73) is mainly expressed in bile duct epithelial cells but hardly expressed in hepatocytes [34]. Serum GP73 levels [34] were elevated in NASH patients and increased with the severity of inflammation, with an AUC of 0.89 for the diagnosis of NASH. Kar et al. [35] found that interleukin-6 (IL-6) and vascular cell adhesion molecule 1 (VCAM-1) could effectively differentiate the severity of NASH, with AUCs of 0.83 and 0.87. IL-8 levels were increased in obese patients with NASH and could be used as a potential diagnostic biomarker [36]. The chemokine C-X-C motif chemokine 10 (CXCL10) was elevated in NASH patients, and the AUC for the diagnosis of SS was 0.81, for NASH was 0.77 [37]. In summary, IL-6, VCAM-1, IL-8, and CXCL10 are potentially inflammatory diagnostic biomarkers for NASH, but these biomarkers are not disease-specific and may be disturbed by systemic inflammation, so a comprehensive clinical judgment is needed. Besides, serum GP73 might become a more specific inflammatory biomarker for NASH, but the diagnostic value needs to be externally validated in multicenter studies.

## 4. Fibrosis Biomarkers

NASH-related fibrosis could progress into hepatic decompensation and end-stage HCC, significantly increasing the risks of liver-related mortality and extrahepatic complications [45]. Therefore, it is necessary to screen for the risk of advanced fibrosis in NAFLD patients before bariatric surgery [46]. PLT are fundamental molecules in the development of fibrosis, and antiplatelet therapy reduced the incidence and mortality of NASH [47], indicating that PLT could be a diagnostic and therapeutic target for NASH. Numerous studies have constructed combined diagnostic models including PLT with high SPE in the diagnostic of progressive fibrosis (Table 3) [10, 32, 45]. Siddiqui et al. [48] evaluated the diagnostic efficacy of the NAFLD fibrosis score (NFS), fibrosis-4 (FIB-4), and AST/platelet ratio index (APRI) for NASH-related fibrosis with C-statistics of 0.80, 0.78, and 0.76 and NPV of 93%, 91%, and 91%, respectively. Udelsman et al. [49] revealed that NFS had better performance in excluding advanced fibrosis with an NPV of 99% in obese individuals. Studies have also reported the best diagnostic performance of the Hepamet fibrosis score (HFS) in advanced fibrosis among NAFLD patients [50]. However, Higuera-de-la-Tijera et al. concluded that HFS had a low positive predictive value of 36.7% in the Mexican population [51]. Importantly, NAFLD/NASH is a pandemic prothrombotic condition strongly associated with CVD risks [50, 52, 53]. Therefore, these noninvasive diagnostic panels of NAFLD severity could also assess the hepatological and cardiometabolic risks in NAFLD patients [50]. Taken together, combined diagnostic panels could be invoked as exclusion indicators for advanced fibrosis, but large-scale validation is still needed [54].

Alpha-2 macroglobulin (A2M), hyaluronic acid (HA), and tissue inhibitor of metalloproteinase-1 (TIMP1) are specific biomarkers of fibrosis. The AUCs for the diagnosis of NASH-related fibrosis were 0.77, 0.81, and 0.78, respectively [55]. Combining these three indicators, the AUC was 0.87 [55]. Furtherly combining HA, procollagen III N-terminal peptide (PIIINP), and TIMP-1 (ELF model), the AUC could reach 0.95, with an SEN of 87% and SPE of 93%, indicating the high clinical application value in the diagnosis of fibrosis, but the model was not sensitive for early fibrosis [56]. Type IV collagen is differentially expressed in patients with NASH and fibrosis, suggesting that it could be a possible biomarker to distinguish NASH from early fibrosis [57]. Type III procollagen (PRO-C3) also showed high expression in fibrosis patients and increased with the degree of fibrosis [58]. Daniels et al. [59] constructed ADAPT score by incorporating age, diabetes, PRO-C3, and PLT, and the AUC for diagnosing progressive fibrosis was 0.86 and was better than the conventional APRI, FIB-4, and NFS scores. Kanno et al. [60] found that aldo-keto reductase family 1 member B10 (AKR1B10) was highly positively correlated with the fibrosis stage and thus could be a possible diagnostic and prognostic biomarker. In conclusion, A2M, HA, TIMP1, PIIINP, type IV collagen, PRO-C3, and AKR1B10 are biomarkers that directly respond to fibrosis in NASH, but these biomarkers remain to be investigated in patients with other chronic liver diseases, and further validation is needed for their extension in the clinical setting.

## 5. Adipokines and Hepatokines

Adipokines and hepatokines [72] are involved in the dialogue between the liver and adipocytes, including adiponectin, visfatin, resistin, adipocyte fatty acid-binding protein (AFABP), angiopoietin-like proteins-1,2,3,4,6,8 (ANGPTL), fetuin-A,B, FGF-1,2,19,21, heparin, and retinol-binding protein (RBP-4) [45]. The expression levels of resistin, visfatin, and RBP-4 did not differ between NASH and SS patients after correction of BMI or waist; therefore, the diagnostic value of these biomarkers alone for NAFLD needs to be further explored [73]. A combined diagnostic panel combining adiponectin, visfatin, TNF-*α*, and IL-6 could effectively differentiate NASH and SS with a SEN of 90% and SPE of 66%[74], suggesting the possible usage as an early screening model for NASH. Fibroblast growth factor-21 (FGF-21) was reported to be increased in obese adolescents and independently correlated with NAFLD [75]. Overall, adipokines and hepatokines were potential diagnostic biomarkers of NAFLD, but the diagnostic accuracy is not yet high enough, and these biomarkers are not yet widely used in clinical practice.

## 6. Omics Biomarkers

### 6.1. Genomics

PNPLA3 rs738409 and TM6SF2 rs58542926 are the most reported NAFLD-related single nucleotide polymorphisms (SNPs) [76]. PNPLA3 and TM6SF2 significantly increased the risk of inflammation and fibrosis progression in NAFLD, even after the correction of insulin resistance [77]. Therefore, they could be used as valid diagnostic biomarkers for NASH. Research also reported that polygenetic risk scores combining PNPLA3, GCKR, and TM6SF2 were significantly associated with an increased risk of NAFLD in obese patients [78]. Hyysalo et al. [79] combined PNPLA3, AST, and insulin to establish the “NASH score” model, and the AUC for the diagnosis of NASH was 0.77. Koo et al. [80] developed the “NASH PT score” with an AUC of 0.86 for the diagnosis of NASH. Therefore, combining genetic information and routine indicators could effectively predict NASH. Other reported NASH-related SNPs included DYSF, MBOAT7, LYPLAL1, PPP1R3B, HSD17B13, PYGO1, and GATAD2A [76, 81], and “NAFLD liver fat score,” “NASH ClinLipMet score,” and “HCC risk score” [82].

### 6.2. Epigenomics

DNA methylation affects the gene expression levels and is associated with the heterogeneity of NAFLD [83]. Research showed that the methylation levels of peroxisome proliferator-activated receptor *γ* (PPAR*γ*) in plasma-free DNA could differentiate the severity of NAFLD and was a potential noninvasive biomarker for NAFLD [84]. It has further been reported that the methylation status of 22 CpG correlated with the degree of steatosis [85]. Hyun et al. [86] detailed and summarized the association of methylation status of genes such as SLC7A11, ACSL4, and CPT1C in peripheral blood with NASH. All in all, these studies suggested that combined diagnostic panels might assist clinicians in diagnosing NASH and predicting disease progression, but further external validation is required.

### 6.3. Transcriptomics

MicroRNA (miRNA) mainly regulates downstream gene expression at the posttranscriptional level. miRNA-122 and miRNA-34a [87] were the most studied in NASH. The AUC of miRNA-122 for the diagnosis of NAFLD was 0.82 and the AUC of miRNA-34a for the diagnosis of NASH was 0.78. Therefore, miRNA-122 and miRNA-34a are reliable diagnostic biomarkers for NASH, but related studies are still in the preliminary stage. The diagnostic value of other miRNAs including miRNA-33, miRNA-192, miRNA-21, miRNA-375, miRNA-221, and miRNA-222 needs to be validated in larger cohorts [88].

Long noncoding RNA (lncRNA) regulates gene expression mainly through chromatin modification, activation/repression of transcriptional enhancers, and targeting miRNAs (ceRNAs) [89]. A study reported that LeXis has an AUC of 0.74, an SEN of 54.3%, and an SPE of 100% for the diagnosis of NASH [89]. Microarray analysis [90] identified lncPRYP4-3 as a potential diagnostic biomarker for NAFLD and revealed its interaction with PRS4Y2. Di Mauro et al. [91] found that the AUC of combined TGFB2/TGFB2-OT1 and FIB-4 was 0.89 for the diagnosis of NASH-related fibrosis. Another study showed that XLOC_014172 and LOC149086 were elevated expressed in patients with HCC and therefore might be used as biomarkers of HCC progression in NASH [92]. Other lncRNAs including FLRL6, FLRL2, lncSTR, lncARS, lnc18q22.2, MEG3, and PVT1 might be potential diagnostic biomarkers for NAFLD [93–95].

CircroRNA- (circRNA-) related studies have focused on its competition with miRNAs to bind target genes and thus regulate downstream gene expression. Overexpression of circRNA_002581 [96] significantly attenuated the inhibitory effect of miR-122 on CPEB1, which is involved in the pathogenesis of NASH through the CPEB1-PTEN-AMPK-mTOR signaling pathway. circRNA_0046367 suppressed the inhibitory effect of miR-34a on PPAR*α*, thereby promoting the expression of downstream lipid metabolism-related genes [97]. circRNA_0046366 expression levels were reduced in NASH patients and highly correlated with oxidative stress, lipotoxicity, and NAFLD disease severity [98], which could be a potential diagnostic biomarker and therapeutic target for NASH. Other circRNAs including circScd1 and circRNA_0071410 were also engaged in the process of lipoatrophy and fibrosis in NAFLD [95].

### 6.4. Proteomics

Yu C et al. [99] identified six biomarkers to establish a combined diagnostic model with 89% SEN and 83% SPE for the diagnosis of NAFLD, and further prospective cohorts found that patients with high levels of hemoglobin were more likely to develop NAFLD, making hemoglobin a potential diagnostic and predictive biomarker for NAFLD. Another study [100] found that ALDOB, APOM, LGALS3BP, PIGR, VTN, and AFM were significantly differentially expressed in NAFLD and control patients by plasma protein profiling, but the clinical application needs to be further investigated. Younossi et al. [101] revealed that A2M and coagulation factor V were highly correlated with NASH-related fibrosis, suggesting that they were potential biomarkers of fibrosis progression.

### 6.5. Metabonomics and Lipidomics

Serum metabolomics revealed that pyroglutamic acid was effective in differentiating patients with NASH and SS with a SEN of 72%, SPE of 85%, and AUC of 0.82 [102]. A study found monounsaturated TG as a specific biomarker for NASH [103]. The AUC for the combination of 11 TGs to distinguish between healthy controls and SS was 0.90, and the combination of 22 TGs to distinguish between SS and NASH was 0.95, suggesting that lipids have a high application value in the diagnosis of NAFLD [104]. Further validating the serum lipid profiles [105], the C16:1n7/C16:0 had an AUC of 0.71 for the diagnosis of NASH and 0.69 for fibrosis patients. A study also found that serum phosphatidylcholine and sphingomyelin levels were markedly increased in SS and NASH patients [106]. Beyoglu and Idle [107] summarized in detail the metabolomic and lipidomic biomarkers associated with NAFLD, including fatty acids, 5-HETE, 8-HETE, 15-HETE, glycyrrhetinic acid, and taurocholate.

Taken together, omics have identified many new NAFLD-related serum biomarkers. It is critical to the early screen obese patients for the risks of NASH and fibrosis progression by these biomarkers (Table 4), while the specific mechanisms and diagnostic value need to be further explored [108]. Due to methodological limitations and reproducibility of results, the identification of omics biomarkers has not yet been widely used in clinical practice.

## 7. Extracellular Vesicles

Extracellular vesicles (EV) are membrane vesicles released by cells, including exosomes, microvesicles, and apoptotic vesicles, which carry a variety of genetic information such as mRNA, noncoding RNA, lipids, and proteins, and are involved in intercellular signaling transduction [109]. EV remains relatively stable in plasma and is, therefore, a reliable diagnostic biomarker for NASH [110]. Lipid-induced damage to hepatocytes significantly increased the release of exosomes [109], which carried TNF-related ligands that interacted with macrophages and induced inflammatory responses. Besides, exosomes derived from hepatocytes could interact with hepatic stellate cells, thus participating in the pathogenesis of progressive fibrosis [109]. Exosomal miRNA-122 is significantly elevated in the NASH model and involved in the macrophage-induced inflammatory response and is, therefore, a reliable diagnostic biomarker for NASH [111, 112]. EV CD14^+^ and EV CD16^+^ significantly increased the accuracy of the ELF model to diagnose severe fibrosis with AUCs of 0.95 and 0.97, respectively [113]. Ban et al. [114] summarized NAFLD-related exosome biomarkers, including CD4, CD8, CD14, CD15, TER119, CD41, CD62P, miRNA-122, and miRNA-192. Shabangu et al. [115] also concluded that cytochrome P450 2E1, toll-like receptor-9, homocysteine, and PPAR*γ* in EV could be used as potential diagnostic biomarkers for NAFLD.

## 8. Conclusions

**Figure 1:**
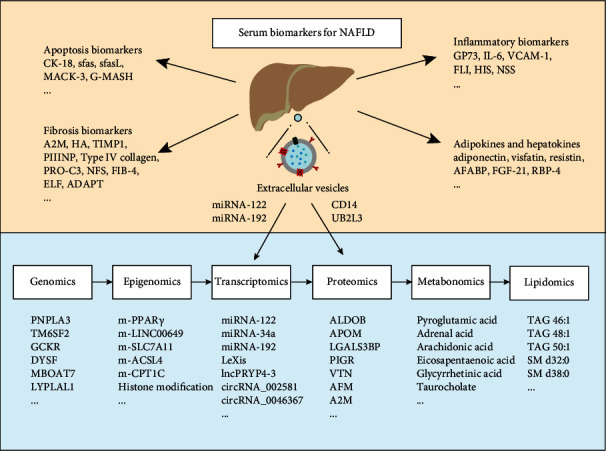
Serum diagnostic biomarkers for NAFLD.

In conclusion, a large number of NAFLD-related serum biomarkers and combination diagnostic panels have been reported (Figure 1). CK-18 is the most studied NASH-related biomarker with high diagnostic efficacy, especially in combination with other biomarkers. The combined diagnostic models of fibrosis such as NFS, FIB-4, and ELF could effectively exclude patients with fibrosis progression, so they could be used as routine screening indicators in clinical practice. Omic biomarkers such as PNPLA3, TM6SF2, miRNA-122, miRNA-34a, and EVs could effectively diagnose patients with NASH, but the practical application still needs further validation. There are no valid and reliable serum biomarkers to differentiate SS and NASH; therefore, it is urgent to explore noninvasive, highly sensitive, highly specific, and clinically accessible biomarkers to identify the severity of NAFLD. Especially in obese patients, it is important to early screen for inflammatory and fibrotic progression of NAFLD and monitor the outcome of bariatric surgery.

## Figures and Tables

**Table 1:** Combined diagnostic panels of apoptosis biomarkers.

Models	Variables	AUC	Diagnostic efficacy	Ref
MACK-3	CK-18 M30, AST, HOMA	NASH: 0.81; fibrosis: 0.80	NASH: cut-off: ≤0.167 and ≥0.551, SEN: 84.2%, SPE: 81.4%; fibrosis: cut-off: ≤0.134, SEN: 100%, SPE: 43.8%	[16]
Nomogram	MACK-3, MS, PLT	Fibrosis: 0.79	Fibrosis: cut-off: ≤137 and ≥180, NPV: 94.7%, SPE: 93.2%	[17]
G-NASH	CK-18 M30, GP73	NASH: 0.85	NASH: accuracy: 62%, non-NASH: accuracy: 100%	[19]
Nice model	CK-18, ALT, MS	NASH: 0.88	NASH: cut-off: 0.14, SEN: 84%, SPE: 86%	[18]
—	CK-18 M30, ALT, PLT, TG	NASH: 0.92	NASH: cut-off: 0.361, SEN: 89%, SPE: 86%	[20]
FIC-22	CK-18 M30, FIB-4	NASH: 0.82; fibrosis: 0.78	NASH: cut-off: 1, SEN: 89.1%, SPE: 62.5%; fibrosis: cut-off: 1, SEN: 87.4%, SPE: 56.1%	[21]
FICK-3	CK-18 M30, FIB-4, HOMA	NAFLD: 0.84; fibrosis: 0.95	—	[22]
NASH diagnostic™	CK-18 M30, adiponectin, resistin	NASH: 0.91	NASH: cut-off: 0.2272, SEN: 95.45%, SPE: 70.21%	[14]
—	CK-18 M65, IL-6, adiponectin	NASH: 0.90	NASH: SEN: 84.5%, SPE: 85.7%	[23]
—	CK-18 M30, FGF-21	NASH: 0.94	NASH: SEN: 92%, SPE: 85%	[24]
CHeK	CK-18 M30, GGT, age, HbA1c, adiponectin	NASH: 0.73	—	[25]
—	CK-18 M30, sFas	NASH: 0.93	NASH: cut-off: -0.5509, SEN: 88%, SPE: 89%	[26]
	sFasL, chemokine 2, race	NASH: 0.71; fibrosis: 0.75	NASH: cut-off: OR = 1.56, SEN: 63.64%, SPE: 86.67%; fibrosis: cut-off: OR = 6.29, SEN: 59.38%, SPE: 80.00%	[27]
—	CK-18 M30, cathepsin D	NASH: 0.998	—	[28]

NAFLD: nonalcoholic fatty liver disease; NASH: nonalcoholic steatohepatitis; AUC: area under the curve; SEN: sensitivity; SPE: specificity; CK-18: cytokeratin-18; AST: aspartate aminotransferase; HOMA: homeostasis model assessment; MS: metabolic syndrome; PLT: platelet; GP73: golgi protein 73; ALT: alanine aminotransferase; TG: triglyceride; FIB-4: fibrosis-4; IL-6: interleukin-6; FGF-21: fibroblast growth factor-21; GGT: *γ*-glutamyl transpeptidase; HbA1c: glycosylated hemoglobin.

**Table 2:** Combined diagnostic models of routine indicators.

Models	Variables	AUC	Diagnostic efficacy	Ref
FLI	BMI, waist, TG, GGT	NAFLD: 0.84	NASH: cut-off: <30 and ≥60, SEN: 87%, SPE: 86%	[38]
HIS	ALT/AST, BMI, gender, diabetes	NAFLD: 0.81	NAFLD: cut-off: <30 and >36, SEN: 92.5%, SPE: 92.4%	[39]
NSS	Age, BMI, TG, FPG, ALT/AST, UA	Men: 0.83; women: 0.86	Men: cut-off: 33, SEN: 79.86%, SPE: 66.13%; women: cut-off: 29, SEN: 89.39%, SPE: 68.98%	[40]
ION	WHR, TG, ALT, HOMA	NAFLD: 0.77	NAFLD: cut-off: <11, SEN: 81%, SPE: 56%; cut-off: ≥22, SEN: 60%, SPE: 82%	[41]
NAFLD liver fat score	MS, diabetes, insulin, AST, AST/ALT	NAFLD: 0.87	NAFLD: cut-off: -0.640, SEN: 86%, SPE: 71%	[42]
HAIR score	HBP, ALT, insulin, GLU	NASH: 0.68	NASH: cut-off: 3, SEN: 57%, SPE: 77%	[43]
SteatoTest-2	A2M, ApoA1, HP, GGT, TC, GLU, ALT, AST, age, gender	SS: 0.68	SS: SEN: 95.5%, PPV: 97.0%	[44]
NASHTest-2	A2M, ApoA1, HP, TBIL, GGT, TC, TG	NASH: 0.59	NASH: SEN: 83.3%, SPE: 37.5%	[44]

NAFLD: nonalcoholic fatty liver disease; NASH: nonalcoholic steatohepatitis; SS: simple steatosis; AUC: area under the curve; SEN: sensitivity; SPE: specificity; BMI: body mass index; TG: triglycerides; GGT: *γ*-glutamyl transpeptidase; ALT: alanine aminotransferase; AST: aspartate aminotransferase; FPG: fasting glucose; UA: uric acid; WHR: waist-hip ratio; HOMA: homeostasis model assessment; MS: metabolic syndrome; HBP: high blood pressure; GLU: glucose; A2M: alpha-2 macroglobulin; APOA1: apolipoprotein A1; HP: haptoglobin; TC: total cholesterol; TBIL: total bilirubin.

**Table 3:** Combined diagnostic panels of fibrosis biomarkers.

Models	Variables	AUC	Diagnostic efficacy	Ref
FibroTest	A2M, APOA1, HP, TBIL, GGT	Advanced fibrosis: 0.79	—	[44]
NFS	Age, BMI, diabetes, AST/ALT, PLT, ALB	Advanced fibrosis: 0.88	Advanced fibrosis: cut-off: <-1.455, SEN: 82%, SPE: 77%; cut-off: >0.676, SEN: 51%, SPE: 98%	[61]
FIB-4	Age, AST, PLT, ALT	Advanced fibrosis: 0.78	Advanced fibrosis: cut-off: <1.3, SEN: 82%, SPE: 57%; cut-off: ≥2.67, SEN: 36%, SPE: 93%	[62]
HFS	Sex, age, diabetes, GLU, INS, HOMA, AST, ALB, PLT	Advanced fibrosis: 0.85	Advanced fibrosis: cut-off: <0.12, SEN: 70.7%, SPE: 80.9%; cut-off: ≥0.47, SEN: 38%, SPE: 98%	[63]
APRI	AST/PLT	Moderate fibrosis: 0.73; advanced fibrosis: 0.76	Moderate fibrosis: cut-off: 0.84, SEN: 65%, SPE: 71%; advanced fibrosis: cut-off: 0.84, SEN: 75%, SPE: 65%	[48]
ARR	AST/ALT	Moderate fibrosis: 0.65; advanced fibrosis: 0.68	Moderate fibrosis: cut-off: 0.81, SEN: 54%, SPE: 68%; advanced fibrosis: cut-off: 0.85, SEN: 54%, SPE: 73%	[48]
BARD score	BMI ≥ 28 kg/m^2^, AST/ALT≥0.8, diabetes	Advanced fibrosis: 0.81	Advanced fibrosis: PPV: 43%, NPV: 96%	[64]
FibroMeter NAFLD	Age, weight, GLU, ALT, AST, PLT, ferritin	Significant fibrosis: 0.94	Significant fibrosis: SEN: 78.5%, SPE: 95.9%	[65]
BAAT score	Age, BMI, ALT, TG	Mild fibrosis: 0.68; advanced fibrosis: 0.62	Mild fibrosis: cut-off: 2.00, SEN: 90.4%, SPE: 35%; advanced fibrosis: cut-off: 2.00, SEN: 94.9%, SPE: 23.8%	[66]
AP index	Age, PLT	Advanced fibrosis: 0.88	—	[67]
CDS	PLT, AST/ALT, INR	Advanced fibrosis: 0.95	Advanced fibrosis: cut-off: 5.00, SEN: 89%, SPE: 90%	[67]
HALT-C model	PLT, AST/ALT, INR	Advanced fibrosis: 0.91	Advanced fibrosis: cut-off: 0.7-0.8, SEN: 89%, SPE: 83%	[67]
Hepascore	Age, gender, TBIL, GGT, A2M, HA	Moderate fibrosis: 0.73; advanced fibrosis: 0.81	Moderate fibrosis: cut-off: 0.44, SEN: 50.5%, SPE: 88.3%; advanced fibrosis: cut-off: 0.37, SEN: 75.5%, SPE: 84.1%	[68]
—	A2M, HA, TIMP1	Advanced fibrosis: 0.87	Advanced fibrosis: cut-off: 17, SEN: 84.8%, SPE: 72.3%	[55]
ELF	HA, PIIINP, TIMP-1	Advanced fibrosis: 0.95	Advanced fibrosis: cut-off: 9.8, SEN: 86.7%, SPE: 92.5%	[56]
FibroMeter^V2G^	AST, urea, PLT, PT, HA, A2M	Advanced fibrosis: 0.80	Advanced fibrosis: cut-off: ≥0.434, SEN: 68.3%, SPE: 75.6%	[69]
CA index	Type IV collagen, AST	NASH: 0.86; fibrosis: 0.92	—	[70]
FM-fibro index	VCAM1, HA	Moderate fibrosis: 0.85; advanced fibrosis: 0.92	—	[70]
FM-fibro index	VCAM1, type IV collagen	Moderate fibrosis: 0.86; advanced fibrosis: 0.92	—	[70]
FM-fibro index	Type IV collagen, HA	Moderate fibrosis: 0.86; advanced fibrosis: 0.91	—	[70]
ADAPT	Age, diabetes, PRO-C3, PLT	Advanced fibrosis: 0.86	Advanced fibrosis: cut-off: >6.3287, PPV: 48.4%, NPV: 96.6%	[59]
FIB-C3	Age, BMI, diabetes, PLT, PRO-C3	Advanced fibrosis: 0.89	Advanced fibrosis: cut-off: >-0.4, SEN: 83%, SPE: 80%	[71]
ABC3D	Age, BMI, diabetes, PLT, PRO-C3	Advanced fibrosis: 0.88	Advanced fibrosis: cut-off: >3, SEN: 77%, SPE: 82%	[71]

NAFLD: nonalcoholic fatty liver disease; NASH: nonalcoholic steatohepatitis; AUC: area under the curve; SEN: sensitivity; SPE: specificity; A2M: alpha-2 macroglobulin; APOA1: apolipoprotein A1; HP: haptoglobin; TBIL: total bilirubin; GGT: *γ*-glutamyl transpeptidase; BMI: body mass index; ALT: alanine aminotransferase; AST: aspartate aminotransferase; PLT: platelet; ALB: albumin; GLU: glucose; TG: triglyceride; INR: international normalized ratio; HA: hyaluronic acid; TIMP1: tissue inhibitor of metalloproteinases-1; PIIINP: procollagen III N-terminal peptide; PT: prothrombin time; VCAM1: vascular cell adhesion molecule 1; PRO-C3: procollagen type III.

**Table 4:** Combined diagnostic models of omics biomarkers.

Models	Variables	AUC	Diagnostic efficacy	Ref
NASH score	PNPLA3, insulin, AST	NASH: 0.77	NASH: cut-off: >-1.054, SEN: 75%, SPE: 74%	[79]
NASH PT score	PNPLA3, TM6SF2, diabetes, AST, HOMA-IR, hsCRP	NASH: 0.86	NASH: cut-off: >-0.785, SEN: 91.0%, SPE: 58.1%	[80]
—	PNPLA3, GCKR, GATAD2A	NASH: 0.65	—	[76]
Extended FLI	PNPLA3, BMI, waist, TG, GGT, 2-hour GLU, 2-hour TG/TG	NAFLD: 0.86	NAFLD: cut-off: ≥60, SEN: 48.6%, SPE: 91.93%	[116]
CI+SNP	Weight, waist, BMI, AST/ALT, TG, FPG, APOC3	NAFLD: 0.90	NAFLD: cut-off: >0.2253, SEN: 86.21%, SPE: 82.23%	[117]
NIS4	miRNA-34a, A2M, YKL-40, HbA1c	NASH: 0.80	NASH: cut-off: <0.36, SEN: 80.8%, SPE: 65.2%; cut-off: ≥0.63, SEN: 45.2%, SPE: 90.4%	[118]
—	miRNA-122, miRNA-192, miRNA-21, CK-18	NASH: 0.83	NASH: cut-off: >0.2253, SEN: 86.21%, SPE: 82.23%	[119]
—	miRNA-122, miRNA-1290, miRNA-192, miRNA-27b	NAFLD: 0.86	NAFLD: SEN: 85.55%, SPE: 73.3%	[120]
—	TGFB2/TGFB2-OT1, FIB-4	Advanced fibrosis: 0.89	Advanced fibrosis: SEN: 80%, SPE: 87.5%	[91]
—	TGFB2/TGFB2-OT1, LSM	Advanced fibrosis: 0.89	Advanced fibrosis: SEN: 80%, SPE: 90.6%	[91]
—	m/z: 2760, 2957, 2967, 5814, 6306, 15, 124 Da	—	SEN: 89%, SPE: 83%	[99]
—	N184_A3G3F1S3+AFP/N241_A3G3F1S3+AFP	HCC: 0.84	HCC: cut-off: 2.25, SEN: 70%, SPE: 83%; cut-off: 2.75, SEN: 70%, SPE: 87%	[121]
GlycoNASHTest	Log(NGA2F/NA2)	NASH: 0.74; advanced fibrosis: 0.87	Advanced fibrosis: SEN: 89.5%, SPE: 71.4%	[122]
—	11 TGs	SS: 0.90	SS: SEN: 98%, SPE: 78%	[104]
—	29 TGs	NASH: 0.96	NASH: SEN: 91%, SPE: 95%	[123]
—	TAG 46:1, TAG 48:1, TAG 50:1, SM d32:0, SM d38:0	Nonobesity NAFLD: 0.92; NASH: 0.81	—	[124]
—	DAG 34:1, DAG 40:7, DAG 40:8, TAG 46:1, TAG 48:1, TAG 50:2, SM d36:0	Obesity NAFLD: 0.97; NASH: 0.81	—	[124]
—	5-HETE, 7,17-DHDPA, adrenal acid, arachidonic acid, eicosapentaenoic acid, 16-HDOHE, 9-HODE	Mild fibrosis: 0.74	—	[125]
—	BMI, age, gender, ALT, TAG	NASH: 0.83	—	[126]
—	EV CD14+, HA, PIIINP, TIMP-1	Advanced fibrosis: 0.95	Advanced fibrosis: cut-off: -0.8687, SEN: 88.9%, SPE: 94.1%	[113]
—	EV CD16+, HA, PIIINP, TIMP-1	Advanced fibrosis: 0.97	Advanced fibrosis: cut-off: -0.3435, SEN: 88.9%, SPE: 88.2%	[113]
—	EV UB2L3, EV Fas	NASH: 0.77	NASH: SEN: 75%, SPE: 83%	[127]

NAFLD: nonalcoholic fatty liver disease; NASH: nonalcoholic steatohepatitis; AUC: area under the curve; SEN: sensitivity; SPE: specificity; AST: aspartate aminotransferase; HOMR: homeostasis model assessment; hsCRP: high-sensitivity C-reactive protein; TG: triglycerides; GGT: *γ*-glutamyl transpeptidase; GLU: glucose; BMI: body mass index; ALT alanine aminotransferase. FPG: fasting glucose; A2M: alpha-2 macroglobulin; HbA1c: glycated hemoglobin; TAG: triacylglycerol; DAG: diacylglycerol; DM: sphingomyelin; CK-18: cytokeratin-18; AFP: alpha-fetoprotein; HA: hyaluronic acid; TIMP1: tissue inhibitor of metalloproteinases-1; PIIINP: procollagen III N-terminal peptide; EV: extracellular vesicle.

## Data Availability

No data were used to support this study.
